# Effects of crown-root angle on stress distribution in the maxillary central incisors’ PDL during application of intrusive and retraction forces: a three-dimensional finite element analysis

**DOI:** 10.1186/2196-1042-14-26

**Published:** 2013-09-11

**Authors:** Farzin Heravi, Soheil Salari, Behrad Tanbakuchi, Shaghayegh Loh, Motahare Amiri

**Affiliations:** Department of Orthodontics, Mashhad University of Medical Sciences, Azadi Square, Mashhad, 91866 Iran; Private Practice, MSc, Tehran, Iran; Department of Pediatric Dentistry, Islamic Azad University, 9th Neyestan St, Tehran, 19944 Iran; MSc, Yazd, Iran

**Keywords:** Crown-root angle, Finite element method, Stress distribution, Intrusive force, Retraction force, Periodontal ligament, Class II malocclusion treatments

## Abstract

**Background:**

Different crown-root angulations of maxillary central incisors can be assumed as a potential reason for many underscored outcomes of orthodontic treatments. The aim of this study was to determine the effects of the different crown-root angles on stress distributions in the maxillary central incisor’s *periodontal ligament* (PDL) during application of intrusive and retraction forces using a 3D finite element method.

**Methods:**

Two models of a maxillary central incisor were constructed using ANSYS software: the first one with an angle of 166.7° (as a sample of the maxillary central incisor in a class II, division 2 patient) and the other one with an angle of 173.4° (normal angulation). Each of the samples was loaded twice by an intrusive force (0.25 N) and a retraction force (0.5 N) through the ideal position of brackets.

**Results:**

FEM results showed little difference between stress distributions in the two models during intrusion (ten thousandth) compared to retraction (thousandth). In the application of retraction force, the stress concentration on the curved tooth was less than the other.

**Conclusion:**

To produce similar patterns of stress in the PDL, orthodontists can apply 1.18 times heavier retraction forces on the maxillary central incisors in class II, division 2 patients compared to class I patients.

## Background

The importance of tooth morphology in dental treatments has been widely emphasized. The maxillary central incisors are the most visible teeth during unstrained facial activities [1]. They are also the most representatives of the mold design of the teeth and can be easily distinguished from the other teeth in oral cavity [2].

One of the most important aspects of tooth morphology is the axial inclination of the tooth. The correlation between axial inclination of the anterior teeth and the reference planes in cephalometrics has been of significant importance through years. Crowns of the teeth may tilt lingually or labially and this coronal inclination has been vigorously discussed in the literature [3, 4]. In class II, division 2 malocclusions, the crowns of the maxillary central incisors tend to bend lingually, and this abnormal inclination may play a role in developing deep bite in these patients [5]. Abnormal axial inclination of the maxillary central incisors may lead to deep bite appearance [6].

The crown-root angles of maxillary incisors in class II, division 2 malocclusions are significantly different from the other groups of malocclusions [7]. These differences are as follows: shorter roots, larger crowns, greater axial curvatures, and reduced labiopalatal thickness. It is concluded that these severely retracted incisors with abnormal crown-root angles may complicate orthodontic treatments (e.g., limit the amount of palatal root torque needed) [7].

In 1983, Williams and co-workers [8] traced the maxillary central incisors of different malocclusions on the radiographs. They found that the crown-root angles significantly differs between class II, division 2 and class II, division 1 malocclusions. The amounts for class III malocclusions came between these two.

Finite element analysis (FEA) was first introduced by Richard Cournat [9] in 1943. It is a numerical method for analyzing the interaction between materials and forces and the pattern of stress distribution in a given mass. It is well capable of simulating different bodies in different situations.

Weinstein et al. [10] were the first ones to use FEA in dentistry and soon it became popular in the profession, especially in implant studies. In 1980, Takahashi et al. [11] used FEA to evaluate the center of rotation and the stress generated in *periodontal ligament* (PDL) and other supporting tissues. They concluded that the support from PDL was the highest near the cervix followed by the apical one third.

Wilson and colleagues [12] applied vertical intrusive and extrusive forces to a 3D model of canine and evaluated the stress established in PDL. The maximum amount of stress was found in alveolar crest. Geramy [13], in a finite element study, showed that the more resorption may result in greater incisal displacement of the apex.

Choy et al. [14] evaluated the effect of bone and root morphology on the stress distribution in PDL, using finite element methods (FEM). They concluded that the morphological variations of the roots have a significant impact on the location of the center of resistance.

In a finite element (FE) study in 2009, Cattaneo et al. [15] evaluated the strain generated in PDL and alveolar bone during orthodontic tooth movements. They found out that (1) in contrary to theoretic science, the tension and pressure sites are not symmetric; and 2) because of the individualized morphology of the bone and roots, the light continuous force will change to an intermittent one.

Liang et al. [16] constructed a finite element model of the maxilla and the maxillary incisors. After loading orthodontic forces from labial and lingual surfaces, they concluded that in order to achieve the best orthodontic results, lingual orthodontics should not simply follow the clinical experience of the labial techniques, but should increase lingual root torque and vertical intrusive force and decrease horizontal retraction force.

Considering the anatomy of the teeth in treating different malocclusions is of great importance. For example, the maxillary central incisor has different crown- root angles in different malocclusions [5, 7]. A constant force applied to anatomically different teeth, will result in different movements.

The aim of the present study was to evaluate the influence of crown- root angle on the stress distribution in the maxillary central incisor’s PDL during the application of retraction and intrusive forces.

## Methods

In the current study, two maxillary central incisors were modeled: one with a low crown-root angle (166.7°) and one with a normal crown-root angle (173.4°). The first one resembled a class II, division 2 malocclusion, and the second one resembled a class I malocclusion on the basis of previous studies [4–17]. The crown length was considered to be 11.2 mm and the root length of teeth was considered to be 13 mm, and the other dimensions such as the crown-root ratio and crown-MD width ratio of the teeth came from a dental anatomy textbook [18]. The thickness of PDL was considered to be 0.25 mm and this thickness was constant. The position and axial inclination of teeth were considered on the basis of ideal occlusion of Andrews [19], and all the elements contributing in the model were assumed to be homogenous. The mechanical properties assigned to the elements were linear-elastic, and Poisson’s ratio was considered 0.3 for all of them. Three different Young’s moduli were chosen to represent bone (12,000 MPa) and PDL (0.05) and tooth structure (20,000 MPa) [20].

**Figure 1:**
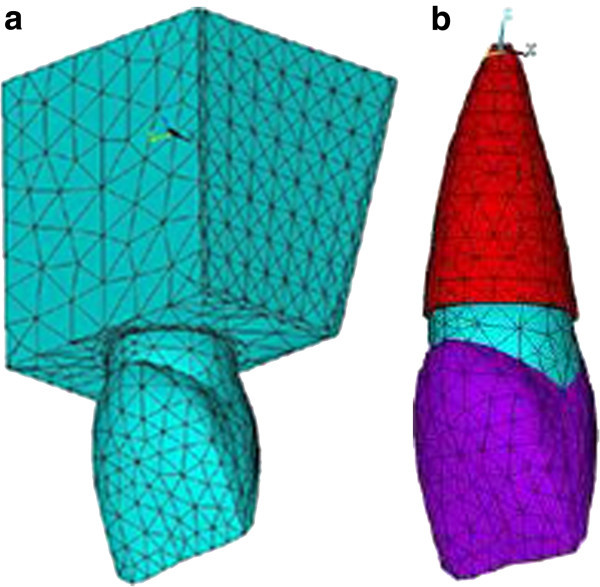
**Model nodes of upper central and surrounding bone and their elements.** **(a)** The elements of upper central and surrounding bone. **(b)** The elements of upper central and its PDL.

The object to be studied was graphically simulated in a computer in the form of a mesh that defined its geometry. In a process called discretization, this mesh was divided into a number of subunits termed elements, which were connected at a finite number of points called nodes. The size of the elements determines the accuracy of the calculations. Therefore, in periodontal ligament area, where the interpretation of the results was of prime importance for us, we chose smaller elements. Each model had 23,000 nodes and 15,700 elements (Figure 1). The analysis was performed with ANSYS software (version 5.4, ANSYS Inc., Canonsburg, PA, USA).

In the present study, force loading was divided into four categories: (1 and 2) intrusive force to the incisors with crown-root angles of 166.7° and 173.4°; (3 and 4) retraction force to the incisors with crown-root angles of 166.7° and 173.4°.

An intrusive force of 0.25 N was loaded at the location of an imaginary bracket (4 mm from the incisal edge) and parallel to the labial surface of the teeth. A retraction force of 0.5 N was also loaded palatally at the same point perpendicular to the labial surface of the teeth. In order to find an equivalent amount of force resembling the same pattern of stress in the PDL of class II, division II patients’ teeth, different levels of retraction force (0.53, 0.56, 0.59, and 0.62 N) were loaded in the form of a trial and error experiment. These increased retraction forces helped us to obtain an idea in the clinical viewpoint.

Stress in the bodies can express itself in different modes: compressive (negative) or tensional (positive). There are a variety of methods for assessing the pattern of loading. The adding up the absolute values of the stresses (along *X*-, *Y*, and *Z*-axes) is known as Von Mises stress [21]. We used this norm to evaluate the pattern of stress generated.

## Results

In this study, we evaluated mainly the stress distribution in PDL which is assumed to be the biologic connector for tooth movements.

The retraction force of 0.5 N and intrusive force of 0.25 N were loaded perpendicularly and parallel to the teeth, respectively. These forces were used at 4-mm distance from the incisal edge. The stresses generated are available in the Tables 1 and 2.

**Table 1:** **Stress in two points from the models of maxillary central incisors during retraction force loading**

Node	CRA 166.7°	Stress in C_1_	CRA 173.4°	Stress in C_2_	C_1_-C_2_ difference
7,208	B1	0.0118219	B1	0.0152682	-0.0034462
7,533	B2	0.0011850	B2	0.0021201	-0.0009350
7,807	B3	0.0101753	B3	0.0134645	-0.0032891
7,195	P1	0.0096988	P1	0.0135697	-0.0038709
7,502	P2	0.0033937	P2	0.0037428	-0.0003491
7,763	P3	0.0112585	P3	0.0143661	-0.0031076
7,185	A	0.0049120	A	0.0065409	-0.0016289

A, apex; B_1_, a node in the labial surface, near the apex; B_2_, a node in the labial surface, near the middle of the root; B_3_, a node in the labial surface, near the cervix; CRA, crown-root angle; P_1_, a node in the palatal surface, near the apex; P_2_, a node in the palatal surface, near the middle of the root; P_3_, a node in the palatal surface, near the cervix. Stress in the two points from the models of maxillary central incisors with crown-root angles of 166.7° and 173.4° during retraction force loading and the differences between these corresponding points.

**Table 2:** **Stress in two points from the models of maxillary central incisors during intrusive force loading**

Node	CR 166.7°	Stress in C_1_	CRA 173.4°	Stress in C_2_	C_1_-C_2_ difference
7,208	B1	0.0044615	B1	0.0039134	0.0005481
7,533	B2	0.0017597	B2	0.0017262	0.0000334
7,807	B3	0.0051819	B3	0.0047076	0.0004742
7,195	P1	0.0057442	P1	0.0053448	0.0003994
7,502	P2	0.0006166	P2	0.0006907	-0.0000740
7,763	P3	0.0043620	P3	0.0037993	0.0005628
7,185	A	0.0023313	A	0.0021130	0.0002182

A, apex; B_1_, a node in the labial surface, near the apex; B_2_, a node in the labial surface, near the middle of the root; B_3_, a node in the labial surface, near the cervix; CRA, crown-root angle; P_1_, a node in the palatal surface, near the apex; P_2_, a node in the palatal surface, near the middle of the root; P_3_, a node in the palatal surface, near the cervix. Stress in two points from the models of maxillary central incisors with crown-root angles of 166.7° and 173.4° during intrusive force loading and the differences between these corresponding points.

**Figure 2:**
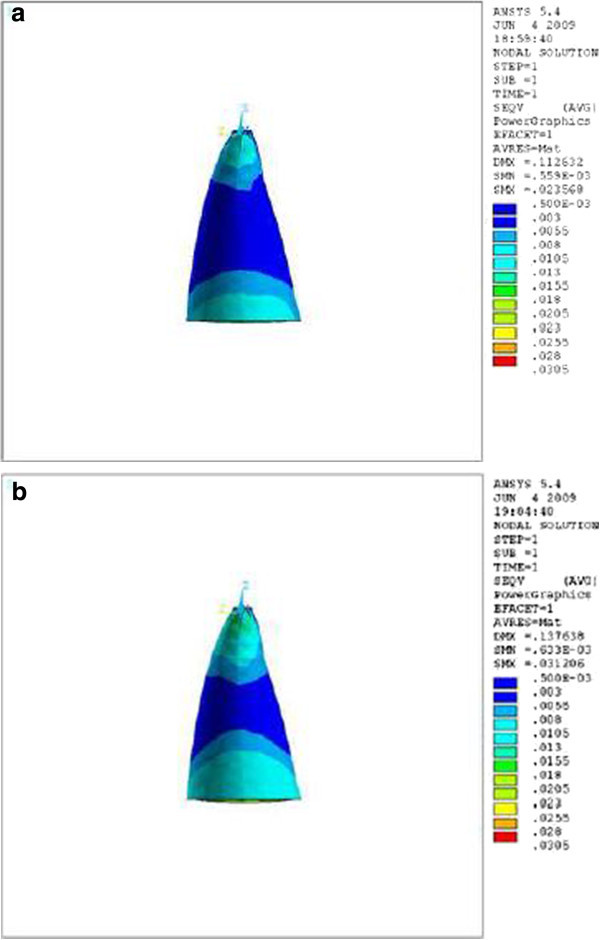
**Stress distribution in PDL of upper central during application of retraction force, labial view.** **(a)** Stress distribution in PDL of upper central with crown-root angle of 166.7° during application of retraction force, labial view. **(b)** Stress distribution in PDL of upper central with crown-root angle of 173.4° during application of retraction force, labial view.

**Figure 3:**
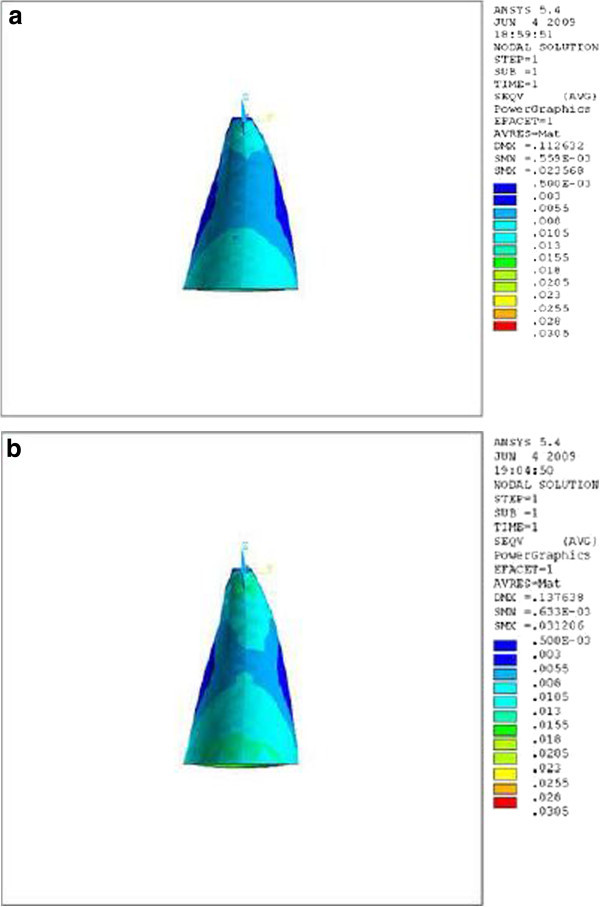
**Stress distribution in PDL of upper central during application of retraction force, palatal view.** **(a)** Stress distribution in PDL of upper central with crown-root angle of 166.7° during application of retraction force, palatal view. **(b)** Stress distribution in PDL of upper central with crown-root angle of 173.4° during application of retraction force, palatal view.

**Figure 4:**
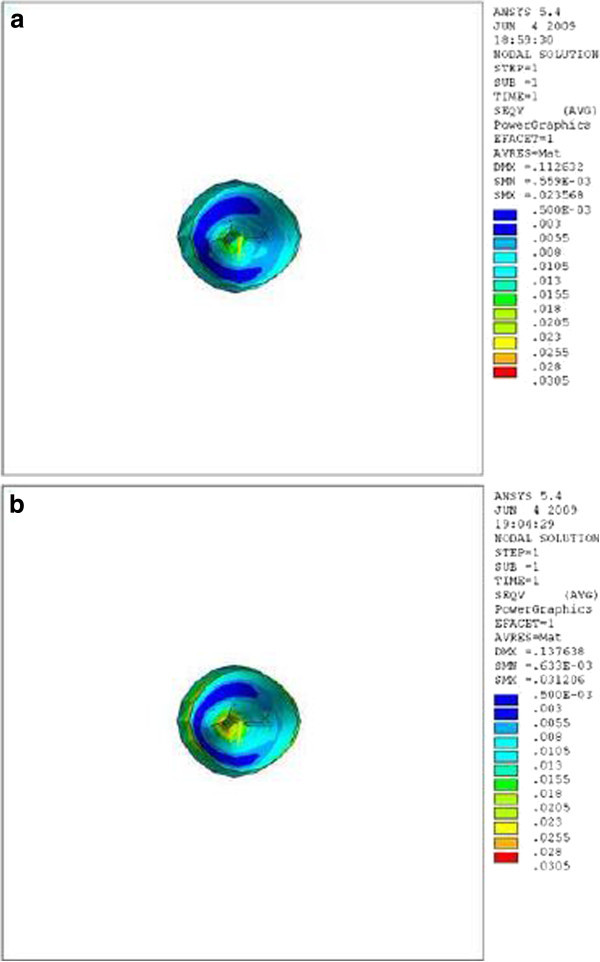
**Stress distribution in PDL of upper central during application of retraction force, apical view.** **(a)** Stress distribution in PDL of upper central with crown-root angle of 166.7° during application of retraction force, apical view. **(b)** Stress distribution in PDL of upper central with crown-root angle of 173.4° during application of retraction force, apical view.

Observing the labial and palatal surfaces during the application of retraction force, we found that the stress distribution near the apex and cervix was lower in the tooth with the crown-root angle of 166.7° (Table 1 and Figures 2,3,4). The differences were at the level of thousandths.

**Figure 5:**
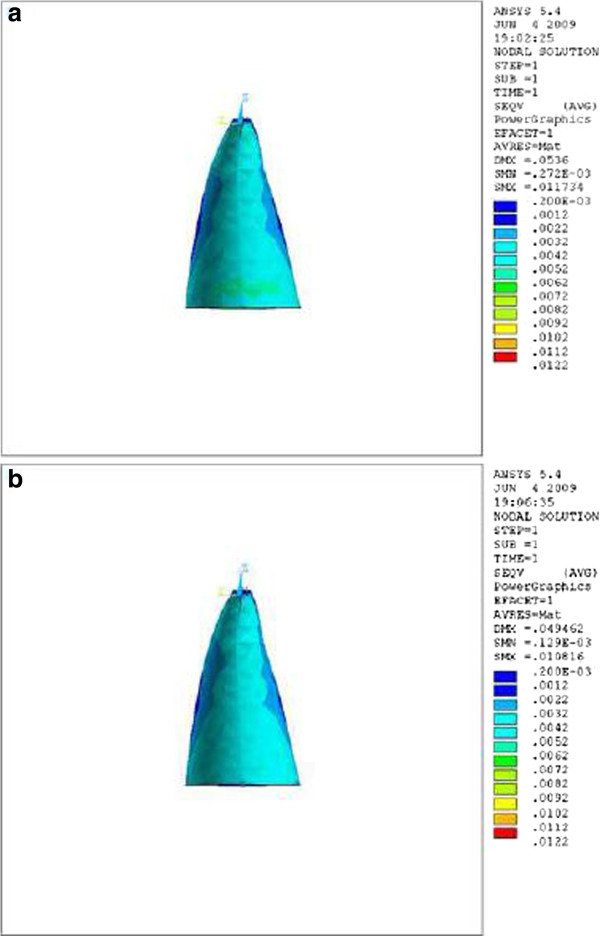
**Stress distribution in PDL of upper central during application of intrusive force, labial view.** **(a)** Stress distribution in PDL of upper central with crown-root angle of 166.7° during application of intrusive force, labial view. **(b)** Stress distribution in PDL of upper central with crown-root angle of 173.4° during application of intrusive force, labial view.

**Figure 6:**
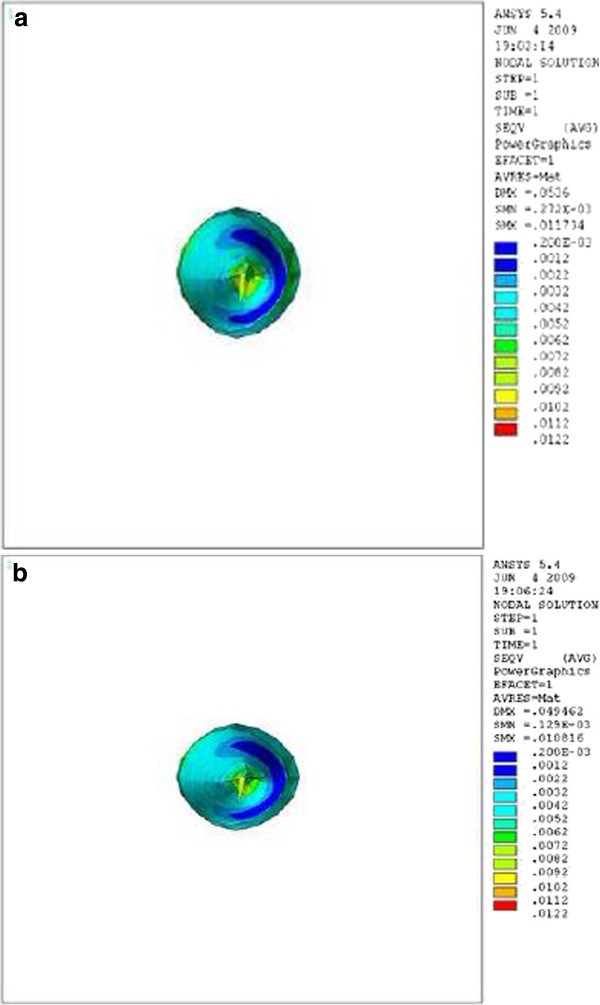
**Stress distribution in PDL of upper central during application of intrusive force, apical view.** **(a)** Stress distribution in PDL of upper central with crown-root angle of 166.7° during application of intrusive force, apical view. **(b)** Stress distribution in PDL of upper central with crown-root angle of 173.4° during application of intrusive force, apical view.

**Figure 7:**
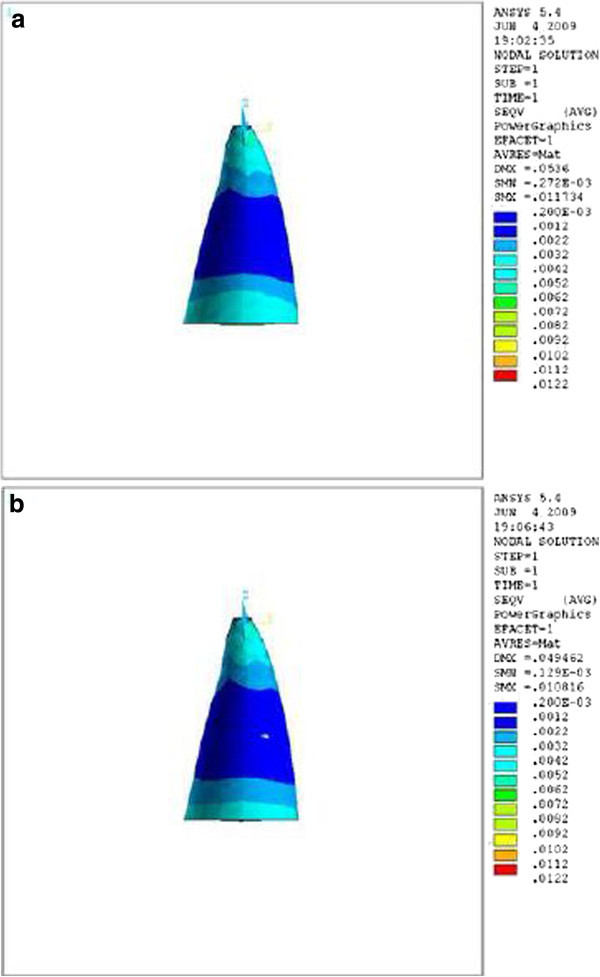
**Stress distribution in PDL of upper central during application of intrusive force, palatal view.** **(a)** Stress distribution in PDL of upper central with crown-root angle of 166.7° during application of intrusive force, palatal view. **(b)** Stress distribution in PDL of upper central with crown-root angle of 173.4° during application of intrusive force, palatal view.

During the application of intrusive force, the stress distribution at the same areas was lower in the tooth with a crown-root angle of 173.4° (Figures 5,6,7), but the differences were at the level of ten thousandths (Table 2).

We increased the amount of retraction force incrementally (0.53, 0.56, 0.59, and 0.62 N) on the model of the smaller crown-root angle, and we found that a retraction force of 0.59 N is almost equally resembles the same pattern of stress distribution as the model with larger angle (Table 3).

**Table 3:** **Stress in three points of maxillary central incisor model during retraction force loading of 59 g**

CRA 166.7°	Node	S1	S2	S3	Stress
B1	7,208	**-9.08E-03**	**-9.57E-03**	**-2.32E-02**	**0.0154186**
B2	7,533	**1.94E-03**	**-2.33E-04**	**-2.73E-03**	**0.0019375**
B3	7,807	**2.07E-02**	**8.69E-03**	**8.22E-03**	**0.0137846**
P1	7,195	**2.08E-02**	**8.07E-03**	**6.48E-03**	**0.0134312**
P2	7,502	**3.76E-03**	**-6.17E-04**	**-5.81E-03**	**0.0040120**
P3	7,763	**-7.38E-03**	**-9.02E-03**	**-2.27E-02**	**0.0147424**
A	7,185	**7.49E-03**	**-1.16E-04**	**-8.58E-03**	**0.0065767**

A, apex; B_1_, a node in the labial surface, near the apex; B_2_, a node in the labial surface, near the middle of the root; B_3_, a node in the labial surface, near the cervix; CRA, crown-root angle; P_1_, a node in the palatal surface, near the apex; P_2_, a node in the palatal surface, near the middle of the root; P_3_, a node in the palatal surface, near the cervix. Stress in three points of the maxillary central incisor model with crown-root angle of 166.7° during retraction force loading of 59 g.

## Discussion

*In vitro* studies provided the orthodontists with new concepts on the behavior of the oral and dental tissues in response to the forces [11, 22, 23]. Results from FEM are highly reliable [16, 23]. Although it is not possible to exactly simulate the *in vivo* conditions such as blood pressure, cellular responses, pH, and oxygen pressure, FEM may shed lights on some unknown aspects of tooth movement (e.g., response of PDL to orthodontic forces). Proffit [24] pointed out that the change in PDL is the first and key biochemical phenomenon of tooth movement. It is feasible to partially anticipate this movement by the help of FEM. The current study was a three-dimensional analysis of stress distribution in PDL.

The abnormal axial inclination of maxillary central incisors in patients with class II, division 2 malocclusions is thought to play an important role in the development of deep bite [6]. There are significant differences in the crown-root angles of maxillary central incisors among different malocclusions [7, 8]. These teeth have a more curved axis in comparison to the other groups. The crown-root angle of maxillary central incisor in class II, division 2 malocclusion differs significantly from the others.

Liang et al. [16] concluded in their study that applying a combination of labial retraction and intrusive force accompanying a counterclockwise moment to a maxillary incisor resulted in true intrusion. In the other study, Lombardo et al. [25] mentioned that applying labial intrusive force to a mandibular incisor generated labial tipping, but the usage of lingual intrusive force was more effective.

Theoretically, while applying a retraction force of 0.5 N palatally (4 mm away from the incisal edge and perpendicular to the labial surface of the tooth), the vertical distance between the point of force application and the center of resistance of the tooth (an imaginary point between the median and cervical third of the root) in the model with the crown-root angle of 166.7° is less than the model with 173.4°. Therefore, less moment is generated and less stress is also expected in the PDL of the former model. As we can see in Table 1 and Figures 2,3,4, the FE analysis showed the same result. This difference was more significant at the apical and cervical areas. During the application of retraction force, the differences in the stress of the corresponding points are at the level of thousands (Table 1).

But what is the equivalent force for retracting a maxillary central incisor in the class II, division 2 patient? In order to find the answer, different amounts of retraction force were applied to the tooth with the smaller crown-root angle in the form of a trial and error experiment.

When applying a 0.59-N force, the contours of the stress were typically similar to the model with the larger crown-root angle that received 0.5 N of the force. This means that while retracting the maxillary central incisor in class II, division 2 patient, the equivalent optimal force can be assumed and practically loaded 1.18 times heavier than in class I patient (Tables 1,2,3).

After applying the intrusive force to the teeth with different crown-root angles, the forces will divide into different components. The horizontal component in the model with 166.7° angle is larger than the model with 173.4° angle. The FE analysis showed the same. The stress in the buccal and lingual surfaces, especially in the apical and cervical areas of the root, was higher in the model with crown-root angle of 166.7° than the model with 173.4° (Table 2, Figures 5,6,7). The differences in the stress of the corresponding points are at the level of ten thousandths, and these little diversities make no important difference in the amount of the optimal force.

## Conclusions

Two fundamental points can be concluded:

1. When the retraction force is applied, the difference in the stress generated in the PDL of maxillary central incisors with different crown-root angles is at the level of thousandths and it is lower in the model with smaller angle. While retracting maxillary central incisors in class II, division 2 patient, the equivalent force can be 1.18 times heavier than in class I patient.
2. While applying the intrusive force, the difference in the stress generated in the PDL of maxillary central incisors with different crown-root angles is small (at the level of ten thousandths), and it is lower in the model with larger crown-root angle.
